# Ethyl 1-cyclo­hexyl-5-(4-meth­oxy­phen­yl)-1*H*-pyrazole-4-carboxyl­ate

**DOI:** 10.1107/S1600536811049282

**Published:** 2011-11-30

**Authors:** Hoong-Kun Fun, Ching Kheng Quah, B. Chandrakantha, A. M. Isloor, Prakash Shetty

**Affiliations:** a X-ray Crystallography Unit, School of Physics, Universiti Sains Malaysia, 11800 USM, Penang, Malaysia; b Department of Chemistry, Manipal Institute of Technology, Manipal 576 104, India; c Medicinal Chemistry Division, Department of Chemistry, National Institute of Technology-Karnataka, Surathkal, Mangalore 575 025, India; d Department of Printing, Manipal Institute of Technology, Manipal 576 104, India

## Abstract

In the title compound, C_19_H_24_N_2_O_3_, the benzene ring forms a dihedral angle of 65.34 (7)° with the pyrazole ring. The cyclo­hexane ring adopts a chair conformation. In the crystal, mol­ecules are linked into a inversion dimers by pairs of C—H⋯O hydrogen bonds, generating *R*_2_^2^(22) ring motifs.

## Related literature

For general background to pyrazole derivatives, see: Dhanya *et al.* (2009 ▶); Hall *et al.* (2008 ▶); Isloor *et al.* (2000 ▶, 2009 ▶); Ragavan *et al.* (2010 ▶); Premsai Rai *et al.* (2009 ▶). For bond-length data, see: Allen *et al.* (1987 ▶). For related structures, see: Fun *et al.* (2010*a* ▶,*b* ▶, 2011 ▶). For hydrogen-bond motifs, see: Bernstein *et al.* (1995 ▶). For ring conformations, see: Cremer & Pople (1975 ▶).
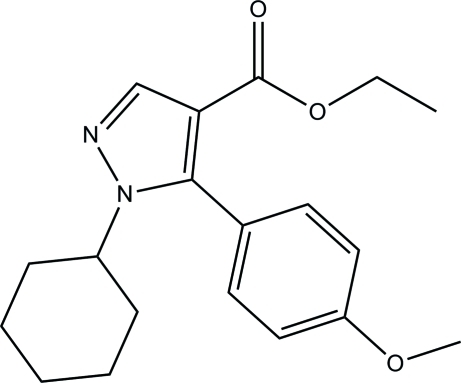


## Experimental

### 

#### Crystal data

C_19_H_24_N_2_O_3_
*M* *_r_* = 328.40
Triclinic, 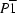

*a* = 6.8959 (7) Å
*b* = 11.0858 (7) Å
*c* = 12.0142 (12) Å
α = 100.690 (2)°
β = 93.107 (1)°
γ = 95.354 (1)°
*V* = 896.16 (14) Å^3^
*Z* = 2
Mo *K*α radiation
μ = 0.08 mm^−1^
*T* = 296 K
0.40 × 0.31 × 0.15 mm

#### Data collection

Bruker SMART APEXII DUO CCD area-detector diffractometer
Absorption correction: multi-scan (*SADABS*; Bruker, 2009 ▶) *T*_min_ = 0.968, *T*_max_ = 0.988
18767 measured reflections
5159 independent reflections
3928 reflections with *I* > 2σ(*I*)
*R*_int_ = 0.021

#### Refinement

*R*[*F* ^2^ > 2σ(*F* ^2^)] = 0.050
*wR*(*F* ^2^) = 0.164
*S* = 1.05
5159 reflections
219 parameters
H-atom parameters constrained
Δρ_max_ = 0.32 e Å^−3^
Δρ_min_ = −0.23 e Å^−3^

### 

Data collection: *APEX2* (Bruker, 2009 ▶); cell refinement: *SAINT* (Bruker, 2009 ▶); data reduction: *SAINT*; program(s) used to solve structure: *SHELXTL* (Sheldrick, 2008 ▶); program(s) used to refine structure: *SHELXTL*; molecular graphics: *SHELXTL*; software used to prepare material for publication: *SHELXTL* and *PLATON* (Spek, 2009 ▶).

## Supplementary Material

Crystal structure: contains datablock(s) global, I. DOI: 10.1107/S1600536811049282/is5014sup1.cif

Structure factors: contains datablock(s) I. DOI: 10.1107/S1600536811049282/is5014Isup2.hkl

Supplementary material file. DOI: 10.1107/S1600536811049282/is5014Isup3.cml

Additional supplementary materials:  crystallographic information; 3D view; checkCIF report

## Figures and Tables

**Table 1:** Hydrogen-bond geometry (Å, °)

*D*—H⋯*A*	*D*—H	H⋯*A*	*D*⋯*A*	*D*—H⋯*A*
C16—H16*A*⋯O2^i^	0.96	2.44	3.358 (2)	159

Symmetry code: (i) 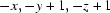.

**Fig. 1.**
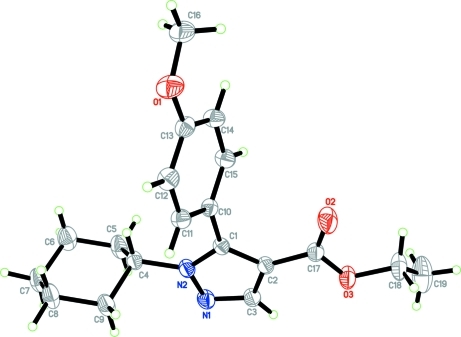
The molecular structure of the title compound, showing 30% probability displacement ellipsoids for non-H atoms.

**Fig. 2.**
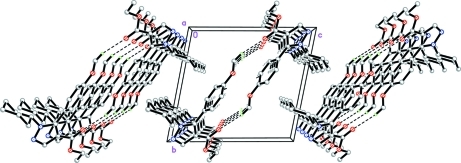
A packing diagram of the title compound, viewed along the a axis. H atoms not involved in hydrogen bonds (dashed lines) have been omitted for clarity.

## supplementary crystallographic information

### Comment

Pyrazoles and their derivatives play an important role in medicinal chemistry (Dhanya *et al.*, 2009). Several derivatives of pyrazole are of pharmaceutical interest due to their analgesic action. Pyrazole molecules also exhibit anticancer (Hall *et al.*, 2008), anti-inflammatory, antidepressant, anticonvulsant and anti-HIV properties (Isloor *et al.*, 2000, 2009). During the past years, considerable evidence has been accumulated to demonstrate the efficacy of pyrazole derivatives. The incorporation of aryl system into the pyrazole ring enhances the biological activities to a great extent (Ragavan *et al.*, 2010). Presence of different substituents, both on the pyrazole ring and on the phenyl ring, can severely modify the biological properties of such molecules (Premsai Rai *et al.*, 2009). Keeping in view of the importance of the pyrazole derivatives, we hereby report the crystal structure of the title compound.

The molecular structure is shown in Fig. 1. The benzene ring (C10–C15) forms a dihedral angle of 65.34 (7)° with the pyrazole ring (N1/N2/C1–C3). Bond lengths (Allen *et al.*, 1987) and angles are within normal ranges and are comparable to related structures (Fun *et al.*, 2010*a*,*b*, 2011). The cyclohexane ring (C4–C9) adopts a chair conformation with puckering parameters (Cremer & Pople, 1975) Q = 0.5694 (17) Å, Θ = 177.82 (16)° and φ = 182 (5)°.

In the crystal (Fig. 2), molecules are linked into an inversion dimer by a pair of intermolecular C16—H16A···O2 hydrogen bonds (Table 1), generating an R^2^_2_(22) ring motif (Bernstein *et al.*, 1995).

### Experimental

A mixture of ethyl 4-methoxy benzoyl acetate (2.0 g, 0.0090 mol) and *N*,*N*-dimethylformamide dimethyl acetal (20 ml) was heated to reflux for 18 h. The excess of acetal was distilled off under reduced pressure and the residue was purified by column chromatography using 60-120 silica gel mesh size with chloroform and methanol as an eluent to give yellow liquid (2.0 g, 95 %). A mixture of ethyl-3-(dimethylamino)-2-(4-methoxyphenylcarbonyl)prop-2-enoate (2.0 g, 0.0088 mol) and cyclohexyl hydrazine (1.1 g, 0.0096 mol) in absolute ethanol (20 ml) was refluxed for 2 h. On cooling, the separated colorless needle-shaped crystals of title compound were collected by filtration. Compound was recrystallized from ethanol (yield 2.5 g, 89%; *m.p.* 413-418 K).

### Refinement

All H atoms were positioned geometrically and refined using a riding model with C—H = 0.93–0.98 Å and *U*_iso_(H) = 1.2 or 1.5*U*_eq_(C). A rotating-group model was applied for the methyl groups.

### Crystal data

**Table:**

C_19_H_24_N_2_O_3_	*Z* = 2
*M**_r_* = 328.40	*F*(000) = 352
Triclinic, *P*1	*D*_x_ = 1.217 Mg m^−^^3^
Hall symbol: -P 1	Mo *K*α radiation, λ = 0.71073 Å
*a* = 6.8959 (7) Å	Cell parameters from 6690 reflections
*b* = 11.0858 (7) Å	θ = 2.8–30.0°
*c* = 12.0142 (12) Å	µ = 0.08 mm^−^^1^
α = 100.690 (2)°	*T* = 296 K
β = 93.107 (1)°	Needle, colourless
γ = 95.354 (1)°	0.40 × 0.31 × 0.15 mm
*V* = 896.16 (14) Å^3^	

### Data collection

**Table:**

Bruker SMART APEXII DUO CCD area-detector diffractometer	5159 independent reflections
Radiation source: fine-focus sealed tube	3928 reflections with *I* > 2σ(*I*)
graphite	*R*_int_ = 0.021
φ and ω scans	θ_max_ = 30.0°, θ_min_ = 1.7°
Absorption correction: multi-scan (*SADABS*; Bruker, 2009)	*h* = −9→9
*T*_min_ = 0.968, *T*_max_ = 0.988	*k* = −15→15
18767 measured reflections	*l* = −16→16

### Refinement

**Table:**

Refinement on *F*^2^	Primary atom site location: structure-invariant direct methods
Least-squares matrix: full	Secondary atom site location: difference Fourier map
*R*[*F*^2^ > 2σ(*F*^2^)] = 0.050	Hydrogen site location: inferred from neighbouring sites
*wR*(*F*^2^) = 0.164	H-atom parameters constrained
*S* = 1.05	*w* = 1/[σ^2^(*F*_o_^2^) + (0.0889*P*)^2^ + 0.118*P*] where *P* = (*F*_o_^2^ + 2*F*_c_^2^)/3
5159 reflections	(Δ/σ)_max_ = 0.001
219 parameters	Δρ_max_ = 0.32 e Å^−^^3^
0 restraints	Δρ_min_ = −0.23 e Å^−^^3^

### Special details

**Table:**

Geometry. All esds (except the esd in the dihedral angle between two l.s. planes) are estimated using the full covariance matrix. The cell esds are taken into account individually in the estimation of esds in distances, angles and torsion angles; correlations between esds in cell parameters are only used when they are defined by crystal symmetry. An approximate (isotropic) treatment of cell esds is used for estimating esds involving l.s. planes.
Refinement. Refinement of F^2^ against ALL reflections. The weighted R-factor wR and goodness of fit S are based on F^2^, conventional R-factors R are based on F, with F set to zero for negative F^2^. The threshold expression of F^2^ > 2sigma(F^2^) is used only for calculating R-factors(gt) etc. and is not relevant to the choice of reflections for refinement. R-factors based on F^2^ are statistically about twice as large as those based on F, and R- factors based on ALL data will be even larger.

### Fractional atomic coordinates and isotropic or equivalent isotropic displacement parameters (Å^2^)

**Table:**

	*x*	*y*	*z*	*U*_iso_*/*U*_eq_	
O1	0.24481 (16)	0.40833 (10)	0.42125 (10)	0.0637 (3)	
O2	−0.1292 (2)	0.87054 (11)	0.39497 (11)	0.0756 (4)	
O3	−0.17512 (15)	1.04017 (9)	0.32770 (9)	0.0579 (3)	
N1	0.20159 (16)	0.93769 (11)	0.08374 (10)	0.0500 (3)	
N2	0.24537 (14)	0.83382 (9)	0.12036 (9)	0.0416 (2)	
C1	0.15217 (15)	0.81617 (10)	0.21339 (9)	0.0366 (2)	
C2	0.04032 (16)	0.91465 (11)	0.23818 (10)	0.0387 (2)	
C3	0.07815 (18)	0.98587 (12)	0.15498 (12)	0.0462 (3)	
H3A	0.0227	1.0583	0.1507	0.055*	
C4	0.37555 (16)	0.75420 (12)	0.05627 (10)	0.0424 (3)	
H4A	0.3759	0.6789	0.0880	0.051*	
C5	0.3032 (2)	0.71696 (17)	−0.06831 (13)	0.0634 (4)	
H5A	0.2978	0.7901	−0.1014	0.076*	
H5B	0.1724	0.6744	−0.0750	0.076*	
C6	0.4396 (2)	0.63269 (18)	−0.13218 (14)	0.0705 (5)	
H6A	0.4338	0.5560	−0.1041	0.085*	
H6B	0.3960	0.6133	−0.2122	0.085*	
C7	0.6475 (2)	0.69171 (17)	−0.11840 (13)	0.0630 (4)	
H7A	0.7311	0.6333	−0.1555	0.076*	
H7B	0.6564	0.7628	−0.1550	0.076*	
C8	0.7178 (2)	0.73191 (17)	0.00499 (13)	0.0624 (4)	
H8A	0.8481	0.7750	0.0109	0.075*	
H8B	0.7248	0.6597	0.0393	0.075*	
C9	0.58264 (18)	0.81614 (15)	0.06897 (13)	0.0562 (4)	
H9A	0.6275	0.8367	0.1488	0.067*	
H9B	0.5857	0.8922	0.0398	0.067*	
C10	0.17434 (16)	0.70935 (10)	0.26810 (10)	0.0371 (2)	
C11	0.35203 (17)	0.69279 (12)	0.32133 (11)	0.0438 (3)	
H11A	0.4596	0.7504	0.3226	0.053*	
C12	0.37068 (19)	0.59210 (12)	0.37225 (11)	0.0481 (3)	
H12A	0.4899	0.5828	0.4080	0.058*	
C13	0.21233 (19)	0.50493 (11)	0.37021 (10)	0.0438 (3)	
C14	0.03437 (19)	0.52023 (12)	0.31811 (12)	0.0475 (3)	
H14A	−0.0729	0.4624	0.3168	0.057*	
C15	0.01701 (17)	0.62194 (12)	0.26809 (11)	0.0463 (3)	
H15A	−0.1030	0.6318	0.2337	0.056*	
C16	0.0922 (3)	0.31069 (15)	0.41146 (16)	0.0671 (4)	
H16A	0.1307	0.2515	0.4552	0.101*	
H16B	0.0667	0.2713	0.3332	0.101*	
H16C	−0.0238	0.3433	0.4394	0.101*	
C17	−0.09192 (17)	0.93662 (11)	0.32911 (10)	0.0424 (3)	
C18	−0.3166 (3)	1.06986 (18)	0.41045 (15)	0.0714 (5)	
H18A	−0.3792	0.9945	0.4285	0.086*	
H18B	−0.2514	1.1194	0.4798	0.086*	
C19	−0.4606 (3)	1.1369 (3)	0.3653 (2)	0.1087 (9)	
H19A	−0.5482	1.1625	0.4225	0.163*	
H19B	−0.5330	1.0848	0.3008	0.163*	
H19C	−0.3971	1.2082	0.3425	0.163*	

### Atomic displacement parameters (Å^2^)

**Table:**

	*U*^11^	*U*^22^	*U*^33^	*U*^12^	*U*^13^	*U*^23^
O1	0.0735 (7)	0.0545 (6)	0.0701 (7)	0.0126 (5)	−0.0038 (5)	0.0302 (5)
O2	0.1033 (9)	0.0716 (7)	0.0721 (7)	0.0383 (6)	0.0486 (7)	0.0396 (6)
O3	0.0665 (6)	0.0570 (6)	0.0602 (6)	0.0274 (5)	0.0281 (5)	0.0204 (5)
N1	0.0516 (6)	0.0506 (6)	0.0567 (7)	0.0129 (5)	0.0173 (5)	0.0252 (5)
N2	0.0395 (5)	0.0447 (5)	0.0454 (5)	0.0100 (4)	0.0123 (4)	0.0157 (4)
C1	0.0346 (5)	0.0399 (5)	0.0369 (5)	0.0044 (4)	0.0046 (4)	0.0108 (4)
C2	0.0374 (5)	0.0404 (5)	0.0406 (6)	0.0067 (4)	0.0065 (4)	0.0114 (4)
C3	0.0460 (6)	0.0441 (6)	0.0541 (7)	0.0108 (5)	0.0121 (5)	0.0186 (5)
C4	0.0385 (5)	0.0477 (6)	0.0432 (6)	0.0088 (4)	0.0110 (4)	0.0103 (5)
C5	0.0411 (6)	0.0889 (11)	0.0523 (8)	0.0073 (7)	−0.0009 (6)	−0.0055 (7)
C6	0.0559 (8)	0.0873 (12)	0.0561 (9)	0.0050 (8)	0.0041 (7)	−0.0169 (8)
C7	0.0519 (7)	0.0856 (11)	0.0487 (8)	0.0122 (7)	0.0155 (6)	0.0000 (7)
C8	0.0382 (6)	0.0885 (11)	0.0556 (8)	0.0132 (6)	0.0064 (5)	−0.0025 (7)
C9	0.0371 (6)	0.0732 (9)	0.0508 (7)	0.0023 (6)	0.0061 (5)	−0.0066 (7)
C10	0.0382 (5)	0.0387 (5)	0.0358 (5)	0.0079 (4)	0.0045 (4)	0.0086 (4)
C11	0.0398 (5)	0.0452 (6)	0.0463 (6)	0.0042 (4)	−0.0009 (5)	0.0098 (5)
C12	0.0456 (6)	0.0515 (7)	0.0485 (7)	0.0114 (5)	−0.0056 (5)	0.0127 (5)
C13	0.0548 (7)	0.0409 (6)	0.0384 (6)	0.0121 (5)	0.0034 (5)	0.0110 (5)
C14	0.0466 (6)	0.0438 (6)	0.0536 (7)	0.0008 (5)	0.0007 (5)	0.0159 (5)
C15	0.0391 (5)	0.0485 (6)	0.0536 (7)	0.0045 (5)	−0.0016 (5)	0.0171 (6)
C16	0.0854 (11)	0.0509 (8)	0.0738 (10)	0.0118 (7)	0.0184 (8)	0.0292 (7)
C17	0.0428 (5)	0.0434 (6)	0.0428 (6)	0.0089 (4)	0.0073 (5)	0.0098 (5)
C18	0.0777 (10)	0.0834 (11)	0.0647 (10)	0.0400 (9)	0.0340 (8)	0.0200 (8)
C19	0.0807 (13)	0.167 (2)	0.0849 (15)	0.0658 (15)	0.0120 (11)	0.0140 (15)

### Geometric parameters (Å, °)

**Table:**

O1—C13	1.3590 (15)	C7—H7B	0.9700
O1—C16	1.421 (2)	C8—C9	1.5183 (19)
O2—C17	1.1962 (16)	C8—H8A	0.9700
O3—C17	1.3328 (15)	C8—H8B	0.9700
O3—C18	1.4463 (17)	C9—H9A	0.9700
N1—C3	1.3178 (16)	C9—H9B	0.9700
N1—N2	1.3598 (14)	C10—C15	1.3856 (16)
N2—C1	1.3551 (14)	C10—C11	1.3945 (16)
N2—C4	1.4657 (15)	C11—C12	1.3814 (17)
C1—C2	1.3917 (15)	C11—H11A	0.9300
C1—C10	1.4729 (15)	C12—C13	1.3849 (18)
C2—C3	1.4048 (16)	C12—H12A	0.9300
C2—C17	1.4611 (16)	C13—C14	1.3862 (18)
C3—H3A	0.9300	C14—C15	1.3840 (17)
C4—C9	1.5128 (17)	C14—H14A	0.9300
C4—C5	1.520 (2)	C15—H15A	0.9300
C4—H4A	0.9800	C16—H16A	0.9600
C5—C6	1.524 (2)	C16—H16B	0.9600
C5—H5A	0.9700	C16—H16C	0.9600
C5—H5B	0.9700	C18—C19	1.436 (3)
C6—C7	1.507 (2)	C18—H18A	0.9700
C6—H6A	0.9700	C18—H18B	0.9700
C6—H6B	0.9700	C19—H19A	0.9600
C7—C8	1.506 (2)	C19—H19B	0.9600
C7—H7A	0.9700	C19—H19C	0.9600
			
C13—O1—C16	117.95 (11)	C4—C9—C8	110.74 (12)
C17—O3—C18	116.56 (11)	C4—C9—H9A	109.5
C3—N1—N2	104.76 (10)	C8—C9—H9A	109.5
C1—N2—N1	112.70 (9)	C4—C9—H9B	109.5
C1—N2—C4	128.17 (10)	C8—C9—H9B	109.5
N1—N2—C4	119.08 (10)	H9A—C9—H9B	108.1
N2—C1—C2	105.63 (10)	C15—C10—C11	117.99 (10)
N2—C1—C10	122.86 (10)	C15—C10—C1	120.39 (10)
C2—C1—C10	131.51 (10)	C11—C10—C1	121.63 (10)
C1—C2—C3	105.00 (10)	C12—C11—C10	120.98 (11)
C1—C2—C17	127.24 (10)	C12—C11—H11A	119.5
C3—C2—C17	127.73 (11)	C10—C11—H11A	119.5
N1—C3—C2	111.91 (11)	C11—C12—C13	120.19 (11)
N1—C3—H3A	124.0	C11—C12—H12A	119.9
C2—C3—H3A	124.0	C13—C12—H12A	119.9
N2—C4—C9	110.98 (10)	O1—C13—C12	116.10 (11)
N2—C4—C5	111.32 (10)	O1—C13—C14	124.31 (12)
C9—C4—C5	110.76 (11)	C12—C13—C14	119.60 (11)
N2—C4—H4A	107.9	C15—C14—C13	119.71 (11)
C9—C4—H4A	107.9	C15—C14—H14A	120.1
C5—C4—H4A	107.9	C13—C14—H14A	120.1
C4—C5—C6	110.07 (12)	C14—C15—C10	121.53 (11)
C4—C5—H5A	109.6	C14—C15—H15A	119.2
C6—C5—H5A	109.6	C10—C15—H15A	119.2
C4—C5—H5B	109.6	O1—C16—H16A	109.5
C6—C5—H5B	109.6	O1—C16—H16B	109.5
H5A—C5—H5B	108.2	H16A—C16—H16B	109.5
C7—C6—C5	111.75 (14)	O1—C16—H16C	109.5
C7—C6—H6A	109.3	H16A—C16—H16C	109.5
C5—C6—H6A	109.3	H16B—C16—H16C	109.5
C7—C6—H6B	109.3	O2—C17—O3	122.85 (12)
C5—C6—H6B	109.3	O2—C17—C2	126.16 (12)
H6A—C6—H6B	107.9	O3—C17—C2	110.95 (10)
C8—C7—C6	111.45 (13)	C19—C18—O3	109.52 (15)
C8—C7—H7A	109.3	C19—C18—H18A	109.8
C6—C7—H7A	109.3	O3—C18—H18A	109.8
C8—C7—H7B	109.3	C19—C18—H18B	109.8
C6—C7—H7B	109.3	O3—C18—H18B	109.8
H7A—C7—H7B	108.0	H18A—C18—H18B	108.2
C7—C8—C9	111.41 (13)	C18—C19—H19A	109.5
C7—C8—H8A	109.3	C18—C19—H19B	109.5
C9—C8—H8A	109.3	H19A—C19—H19B	109.5
C7—C8—H8B	109.3	C18—C19—H19C	109.5
C9—C8—H8B	109.3	H19A—C19—H19C	109.5
H8A—C8—H8B	108.0	H19B—C19—H19C	109.5
			
C3—N1—N2—C1	−0.18 (14)	C7—C8—C9—C4	56.03 (19)
C3—N1—N2—C4	177.65 (11)	N2—C1—C10—C15	−114.31 (13)
N1—N2—C1—C2	0.28 (13)	C2—C1—C10—C15	64.46 (18)
C4—N2—C1—C2	−177.31 (11)	N2—C1—C10—C11	65.81 (16)
N1—N2—C1—C10	179.32 (10)	C2—C1—C10—C11	−115.41 (14)
C4—N2—C1—C10	1.74 (18)	C15—C10—C11—C12	0.16 (19)
N2—C1—C2—C3	−0.25 (13)	C1—C10—C11—C12	−179.97 (11)
C10—C1—C2—C3	−179.18 (12)	C10—C11—C12—C13	0.6 (2)
N2—C1—C2—C17	178.12 (11)	C16—O1—C13—C12	−173.91 (13)
C10—C1—C2—C17	−0.8 (2)	C16—O1—C13—C14	6.2 (2)
N2—N1—C3—C2	0.01 (15)	C11—C12—C13—O1	179.25 (12)
C1—C2—C3—N1	0.15 (15)	C11—C12—C13—C14	−0.9 (2)
C17—C2—C3—N1	−178.20 (12)	O1—C13—C14—C15	−179.74 (12)
C1—N2—C4—C9	−112.62 (14)	C12—C13—C14—C15	0.4 (2)
N1—N2—C4—C9	69.93 (15)	C13—C14—C15—C10	0.4 (2)
C1—N2—C4—C5	123.52 (14)	C11—C10—C15—C14	−0.64 (19)
N1—N2—C4—C5	−53.94 (15)	C1—C10—C15—C14	179.49 (11)
N2—C4—C5—C6	−179.09 (13)	C18—O3—C17—O2	−0.4 (2)
C9—C4—C5—C6	56.92 (18)	C18—O3—C17—C2	177.29 (13)
C4—C5—C6—C7	−55.8 (2)	C1—C2—C17—O2	−2.4 (2)
C5—C6—C7—C8	54.9 (2)	C3—C2—C17—O2	175.59 (15)
C6—C7—C8—C9	−54.7 (2)	C1—C2—C17—O3	−179.98 (11)
N2—C4—C9—C8	178.50 (12)	C3—C2—C17—O3	−1.97 (19)
C5—C4—C9—C8	−57.32 (17)	C17—O3—C18—C19	−149.67 (19)

### Hydrogen-bond geometry (Å, °)

**Table:**

*D*—H···*A*	*D*—H	H···*A*	*D*···*A*	*D*—H···*A*
C16—H16A···O2^i^	0.96	2.44	3.358 (2)	159.

Symmetry codes: (i) −*x*, −*y*+1, −*z*+1.
